# The C Proteins of Human Parainfluenza Virus Type 1 Block IFN Signaling by Binding and Retaining Stat1 in Perinuclear Aggregates at the Late Endosome

**DOI:** 10.1371/journal.pone.0028382

**Published:** 2012-02-15

**Authors:** Henrick Schomacker, Rebecca M. Hebner, Jim Boonyaratanakornkit, Sonja Surman, Emerito Amaro-Carambot, Peter L. Collins, Alexander C. Schmidt

**Affiliations:** RNA Viruses Section, Laboratory of Infectious Diseases, National Institute of Allergy and Infectious Diseases, National Institutes of Health, Bethesda, Maryland, United States of America; University of Georgia, United States of America

## Abstract

Interferons (IFNs) play a crucial role in the antiviral immune response. Whereas the C proteins of wild-type human parainfluenza virus type 1 (WT HPIV1) inhibit both IFN-β induction and signaling, a HPIV1 mutant encoding a single amino acid substitution (F170S) in the C proteins is unable to block either host response. Here, signaling downstream of the type 1 IFN receptor was examined in Vero cells to define at what stage WT HPIV1 can block, and F170S HPIV1 fails to block, IFN signaling. WT HPIV1 inhibited phosphorylation of both Stat1 and Stat2, and this inhibition was only slightly reduced for F170S HPIV1. Degradation of Stat1 or Stat2 was not observed. The HPIV1 C proteins were found to accumulate in the perinuclear space, often forming large granules, and co-localized with Stat1 and the cation-independent mannose 6-phosphate receptor (M6PR) that is a marker for late endosomes. Upon stimulation with IFN-β, both the WT and F170S C proteins remained in the perinuclear space, but only the WT C proteins prevented Stat1 translocation to the nucleus. In addition, WT HPIV1 C proteins, but not F170S C proteins, co-immunoprecipitated both phosphorylated and unphosphorylated Stat1. Our findings suggest that the WT HPIV1 C proteins form a stable complex with Stat1 in perinuclear granules that co-localize with M6PR, and that this direct interaction between the WT HPIV1 C proteins and Stat1 is the basis for the ability of HPIV1 to inhibit IFN signaling. The F170S mutation in HPIV1 C did not prevent perinuclear co-localization with Stat1, but apparently weakened this interaction such that, upon IFN stimulation, Stat1 was translocated to the nucleus to induce an antiviral response.

## Introduction

HPIV1 is the most common cause of croup and is an important respiratory pathogen in young children, the elderly, and the immunocompromised [1], [2], [3]. Although most of the burden of disease in children is treated on an outpatient basis, HPIV serotypes 1, 2, and 3 account for 7% of all hospitalizations for fever and/or acute respiratory illnesses in children under 5 years of age [4]. HPIV infections do not induce complete protection against re-infection, and most of us likely have experienced multiple respiratory illnesses due to HPIVs. However, while host immunity is inefficient in preventing re-infection, it does reduce virus replication and disease during re-infections. The ability of HPIVs to re-infect symptomatically without significant antigenic change is due in part to their tropism to the superficial respiratory epithelium, where the efficiency of immune protection is reduced.

HPIV1 is a Respirovirus in the subfamily Paramyxovirinae, family Paramyxoviridae, order Mononegavirales. Its single strand negative sense RNA genome, 15.6 kb in length, contains 6 genes (3′ – N-P/C-M-F-HN-L – 5′) that encode the nucleoprotein (N), phosphoprotein (P), C proteins (C), matrix protein (M), fusion protein (F), hemagglutinin-neuraminidase protein (HN), and the large polymerase protein (L). Each gene encodes a single protein with the exception of the P/C gene, which encodes the P protein in one open reading frame and a nested set of four carboxy-coterminal C proteins (C′, C, Y1, and Y2, amino acid lengths of 219, 204, 181, and 175, respectively) expressed from individual start sites in a second open reading frame.

Sendai virus (SeV), the most-extensively characterized PIV, is the murine homologue of HPIV1, with considerable sequence relatedness. However, the P/C gene organization of SeV differs from that of HPIV1 in that SeV engages in RNA editing to express, in addition to the C proteins, a second accessory protein called V protein that also inhibits the innate antiviral response as well as having other roles in the replicative cycle [5]. In contrast, HPIV1 does not edit and does not express a V protein. In addition, some of the immune evasion activities of SeV and HPIV1 are species-specific [6], [7], and the two viruses clearly differ in their host range: The lethal dose 50% of some SeV strains is less than 100 infectious units for mice [8], [9] whereas adult humans inoculated with 10^7^ infectious units of SeV do not develop any respiratory illness [10]. In contrast, even high doses of HPIV1 do not cause disease in mice, whereas HPIV1 causes respiratory illness in more than 50% of healthy adults inoculated with less than 100 infectious units of virus [11].

The lack of a V protein sets HPIV1 apart not only from SeV but also from most of the other viruses of the Paramyxovirinae subfamily. With the exception of HPIV1 and HPIV3 – the latter of which either does not express a V protein or does so inefficiently [12], [13] – all members of the Paramyxovirinae subfamily appear to express a V protein. The C-terminal domain of the V protein has a cysteine-rich motif that is highly conserved in Paramyxovirinae [14]. While most members of Paramyxovirinae express a V protein, C proteins are expressed only by members of genus Respirovirus (e.g., HPIV1 and HPIV3), Morbillivirus (e.g., measles virus), and Henipavirus (i.e., Nipah and Hendra viruses). In contrast to the V protein, the C proteins do not have any obvious motifs that are conserved across those viruses that express C.

The phenylalanine 170 to serine (F170S) substitution in the C proteins of SeV was initially discovered because it rendered the mutant virus avirulent in mice [15]. Later it was shown that the SeV C proteins were sufficient to block IFN-β signaling [16], [17] whereas both the C and V proteins have been shown to participate in the inhibition of type 1 IFN induction [7], [18]. The SeV C proteins have also been shown to play roles in the regulation of viral RNA synthesis [19], in virus assembly [20], selective packaging of negative sense RNA genomes into the virion [21], virus budding [22] and inhibition of apoptosis [23]. Some of these activities remain controversial. For example, the report that virion budding depends on interaction between the C protein and a cellular protein called Alix was not confirmed [24]. Also, as noted below, the mechanism(s) by which the SeV C proteins block signaling from the IFN receptor remains unclear.

The HPIV1 C proteins are much less well characterized but have been shown to inhibit apoptosis and IFN-β signaling [6], [25], [26], [27]. We previously transferred the F170S mutation into HPIV1 by reverse genetics, which resulted in a virus that is highly attenuated in non-human primates [15], [25], [26], [28], [29]. Studies with this virus showed that the HPIV1 C proteins regulate and restrain viral RNA synthesis to prevent the formation of dsRNA, thereby indirectly preventing IFN-β induction and activation of protein kinase R [30]. In addition, mutation or deletion of C is associated with changes in the expression of more than 2000 cellular genes compared to WT HPIV1 [27]. Since IFN secretion leads to the establishment of an antiviral state in both infected and non-infected cells, both virus spread and virus replication are restricted [31]. The F170S mutation in HPIV1 is one of the major attenuating mutations in a live HPIV1 vaccine candidate presently in clinical trials (clinicaltrials.gov NCT00641017) [32].

Type 1 IFNs (notably IFN- α and β) and Type 2 IFN (IFN-γ) signal through different receptors, but both types of IFN employ the Jak/Stat signaling pathway [33]. Jak/Stat signaling is initiated by binding of IFN to its transmembrane receptor, which results in the reorganization and auto-phosphorylation of receptor subunits and the binding and phosphorylation of Janus kinases (Jak/Tyk). The Janus kinases then recruit Signal Transducers and Activators of Transcription (Stats) to this membrane-associated complex and phosphorylate them. Phosphorylated Stats then form either Stat1:Stat1 homodimers following IFN-γ stimulation, or Stat1:Stat2 heterodimers and ISGF3 complexes (Stat1:Stat2:IRF9) following type 1 IFN stimulation. These dimers or trimers then translocate to the nucleus where they bind to and activate specific DNA binding sites [34].

The SeV C proteins strongly inhibit signaling from the IFN-α/β receptor, but the mechanism(s) remains unclear and appears to vary with different experimental conditions. One line of experiments provided evidence of ubiquitination and proteosomal degradation of Stat1, an effect that involved the two longer C proteins, C′ and C, but not the shorter Y1 and Y2 forms, and which could be mimicked by the first 23 amino acids of C [17], [35], [36]. Another line of experiments indicated that neither Stat1 nor Stat2 is degraded, and that the C proteins inhibit signaling from the IFN receptor by blocking phosphorylation of both Stat1 and Stat2, with the impaired phosphorylation of Stat2 being the more important effect [37]. The C-terminal 106 residues of C were sufficient to mediate these latter effects [38], and residues 151, 153, and 154, in addition to the F170S mutation, were shown to be important [39]. The inconsistencies in these results may reflect experimental differences such as the use of transfected plasmids or stably-expressing cell lines versus infection, the use of cells from different hosts and in particular from non-host species, and the use of cells that are competent to express type 1 IFN, which can confound results since Stat1 expression is strongly up-regulated by type 1 IFN.

HPIV1 has been shown to inhibit translocation of Stat1 and Stat2 to the nucleus [6], but otherwise the mechanisms by which the HPIV1 C proteins inhibit IFN signaling were unknown. In the present study, we used Vero cells (representing African green monkeys, which are susceptible to HPIV1 infection), which do not express type 1 IFNs and thus permit evaluation of IFN signaling without the confounding effects of endogenously-produced IFN, to examine at what stage in the pathway WT HPIV1 succeeds and F170S HPIV1 fails to inhibit IFN signaling. In addition, we studied these effects mostly in the context of viral infection, since this would provide the most authentic conditions as opposed to transfected cDNAs or stably expressing cell lines that express individual proteins outside of the context of the other viral macromolecules and induced cellular response and with possible differences in expression levels and subcellular distribution. Given the lack of a HPIV1 V protein, the activities of the C proteins can readily be evaluated with fully replication competent viral mutants. One of the findings of this study was co-localization of the C proteins and Stat1 with the cellular protein cation-independent mannose 6-phosphate receptor (M6PR). Mannose 6-phosphate (M6P) is the sorting signal that distinguishes proteins that are destined to reside in the lysosome from those that are destined to be transported to the surface or to be secreted [40]. For proteins destined for the lysosome, N-linked sugars are modified to contain M6P. These proteins are bound by M6PR in the trans-Golgi network and are diverted into clathrin-coated vesicles [41]. These vesicles fuse with endosomes carrying serum proteins ingested at the plasma membrane, creating what are referred to as late endosomes [42]. A small fraction of M6PR also is localized on the cell surface, where it binds to M6P-carrying serum proteins [43], but most of the M6PR is associated with late endosomes, and M6PR is widely accepted as a late endosome marker.

## Results

### In contrast to WT HPIV1, F170S HPIV1 is unable to inhibit IFN-α, -β, or -γ-mediated induction of an antiviral state

**Figure 1:**
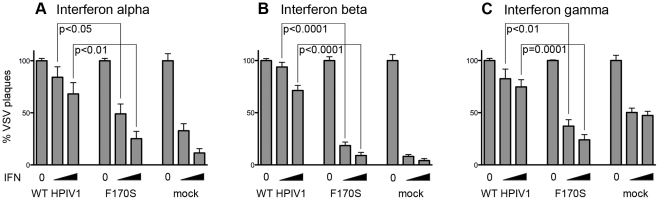
Signaling in WT or F170S HPIV1-infected Vero cells following treatment with IFN-α, -β, or -γ, assayed by VSV-GFP plaque formation. Vero cells were mock-infected or infected with WT or F170S HPIV1 at an MOI of 5 TCID_50_/cell. After 48 h, cells were mock-treated or treated with 100 or 1000 IU/ml of the indicated IFN for 24 h. Subsequently, cells were infected with GFP-expressing VSV, and VSV plaques were enumerated 48 h later. Inhibition of VSV plaque formation is an indication of IFN signaling to create an antiviral state. The relative plaque numbers are shown, as percent of the number of plaques that formed in non-IFN-treated wells. WT and F170S HPIV1-infected cells stimulated with 100 or 1000 IU of any of the three IFNs differed significantly in their ability to restrict VSV plaque formation (P values for two-tailed T-tests are indicated).

We have previously shown that WT HPIV1 is able to inhibit the IFN-β-mediated induction of an antiviral state in human lung A549 cells whereas F170S HPIV1 is unable to do so [25]. The current study sought to better define where in the IFN signaling pathway this block occurred. We examined the Jak/Stat signaling pathway in WT HPIV1- and F170S HPIV1-infected Vero cells following stimulation with IFN-α, -β, or -γ. Vero cells were infected with either virus for 48 h, mock-treated or treated with 100 or 1000 IU of IFN-α, -β or -γ for 24 h, and superinfected with GFP-expressing VSV. Two days later, VSV plaques were enumerated, with inhibition of plaque formation being an indication of IFN signaling and establishment of an antiviral state. As expected, IFN-β treatment induced an antiviral state in mock-infected Vero cells and reduced the number of VSV plaques by up to 97% in a dose-dependent manner (Figure 1). IFN-α also reduced the number of VSV plaques in a dose-dependent manner, as one would expect since IFN-α and IFN-β use the same receptor and signal through Stat1:Stat2 heterodimers. In contrast, IFN-γ treatment, dependent on a different receptor, reduced the number of VSV plaques by no more than 53% (Figure 1). This lower level of inhibition might reflect limited expression of the IFN-γ receptor on Vero cells or a difference in the effectiveness of the cellular antiviral response to type 1 versus type 2 IFN, which activate different sets of genes. For all three IFN treatments, prior WT HPIV1 infection inhibited the IFN-mediated induction of an antiviral state, thereby permitting VSV to form significantly more plaques than in mock-infected Vero cells. In contrast, F170S HPIV1 was unable to inhibit the induction of an IFN-α, -β, or -γ-mediated antiviral state, so that VSV plaque formation was as restricted as in mock-infected cells (Figure 1). The reduced ability of F170S HPIV1 to inhibit the induction of an antiviral state also was reflected in the reduced plaque size of VSV-GFP on cells infected with F170S HPIV1 versus cells infected with WT HIV1, and the reduced expression of GFP (Figure S1).

### Stat1 and Stat2 phosphorylation

**Figure 2:**
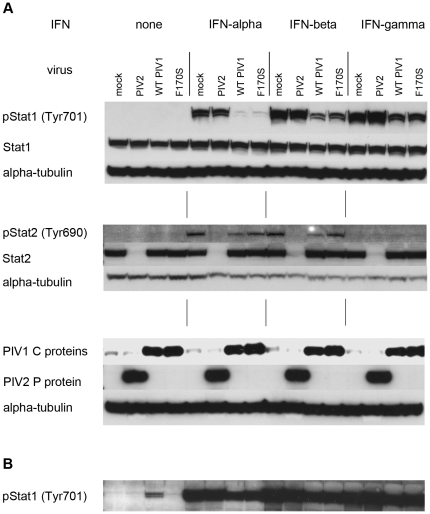
Western blot of total and phosphorylated Stat1 and Stat2 in WT or F170S HPIV1-infected Vero cells following treatment with IFN-α, -β, or -γ. Vero cells were mock-infected or infected with WT HPIV1, F170S HPIV1, or HPIV2 at an MOI of 5 TCID_50_/cell. After 48 h, cells were mock-treated or treated for 30 min with 1000 IU/ml of the indicated IFN. A) Western blots were probed for total or phosphorylated (p)Stat1 and Stat2, as well as for the HPIV1 C protein and HPIV2 P protein. Alpha-tubulin was used as loading control. B) Extended exposure (over night) of the top panel in Figure 2A [“pStat1 (Tyr701)”], showing that a low level of pStat1 is detected in cells infected with WT HPIV1 in the absence of IFN treatment.

Aiming to identify at what step IFN signaling was inhibited by WT but not F170S HPIV1, we analyzed phosphorylation and accumulation of Stat1 and Stat2. We infected Vero cells with either virus for 48 h, mock-treated or treated the cells with 1000 IU/ml of the indicated IFN for 30 min, and subjected cell lysates to Western blot analysis for total and phosphorylated (p) Stat1 and Stat2 (Figure 2). This showed that, following IFN-α or IFN-β treatment, total Stat1 accumulation was unchanged and Stat1 phosphorylation at Tyr701 was reduced in both WT HPIV1- and F170S HPIV1-infected cells (Figure 2). Unexpectedly, there was little difference between the WT and F170S viruses: phosphorylation of Stat1 was only marginally increased for F170S. This lack of difference between the WT and F170S viruses was confirmed by examining multiple time points following IFN-β treatment (Figure S2). Thus, the increase in IFN-α/β signaling observed with F170S HPIV1 did not appear to be due to a loss of the ability to inhibit Stat1 phosphorylation. Interestingly, these results also indicate that the induction of a potent antiviral state is possible (in F170S-infected cells) despite limited Stat1 phosphorylation.

WT or F170S HPIV1 infection also did not result in Stat2 degradation, in contrast to what is seen in HPIV2-infected cells (Figure 2A) [44], [45]. Phosphorylation of Stat2 in response to stimulation with IFN-α or IFN-β was slightly reduced for F170S HPIV1 and somewhat more for WT HPIV1. Again, this difference seemed too small to explain the dramatic increase in IFN-α/β signaling observed with F170S HPIV1. As expected, treatment with IFN-γ did not induce Stat2 phosphorylation, since this is not involved in this signaling pathway.

Interestingly, following longer exposure of the Western blots shown in Figure 2A, a small amount of phosphorylated Stat1 (pStat1) was detected in untreated WT HPIV1-infected cells but not in F170S-infected cells (Figure 2B). One interpretation is that there is a low level of Stat1 phosphorylation/dephosphorylation even in the absence of in IFN-α/β (since Vero cells cannot produce IFN-α/β) that is detectable because WT HPIV1 inhibits dephosphorylation of this low background activity.

In summary, our findings indicate that HPIV1 infection did not lead to Stat1/2 degradation and that phosphorylation of Stat1 and Stat2 was reduced in WT HPIV1- and F170S HPIV1-infected cells following stimulation with IFN-α and IFN-β. However, the extent of Stat phosphorylation did not differ between WT and F170S HPIV1 to an extent that would explain the marked difference in IFN signaling between WT and F170S HPIV1.

### Translocation of Stat1 and Stat2 to the nucleus

**Figure 3:**
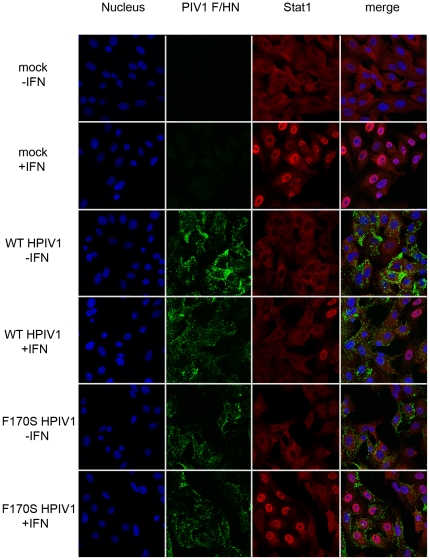
Intracellular localization of Stat1 in WT or F170S HPIV1-infected Vero cells following IFN treatment. Vero cells were mock-infected or infected with WT or F170S HPIV1 at an MOI of 1 TCID_50_/cell, and 48 h later were mock-treated (-IFN) or treated (+IFN) with 1000 IU/ml of IFN-β for 1 h. Cells were fixed, permeabilized, immunostained with antibodies for HPIV1 surface proteins (green) and Stat1 (red), stained with DAPI to visualize nuclei (blue), and analyzed by confocal microscopy. Representative fields are shown. Overall, 2% of the WT HPIV1-infected cells and 82% of the F170S HPIV1-infected cells showed nuclear Stat1 following IFN-β treatment.

**Figure 4:**
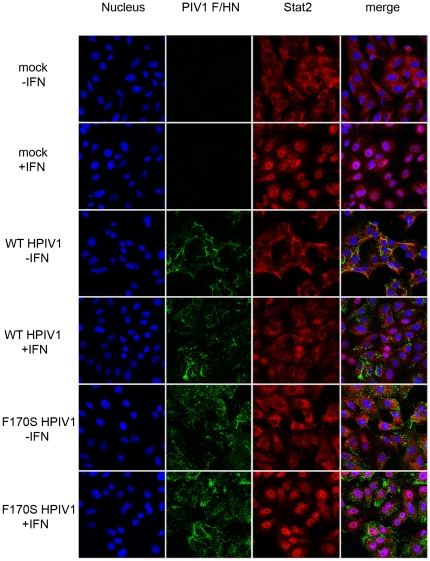
Intracellular localization of Stat2 in WT or F170S HPIV1-infected Vero cells following IFN treatment. Cells were infected and analyzed as described in the legend to Figure 3 except that the antibodies against Stat1 were replaced with antibodies against Stat2 (red). Representative fields are shown. Overall, 2% of the WT HPIV1-infected cells and 100% of the F170S HPIV1-infected cells showed nuclear Stat1 following IFN-β treatment.

Since no significant differences were observed with regard to Stat1 or Stat2 phosphorylation or stability between WT and F170S HPIV1-infected cells, we next examined translocation of Stat1 and Stat2 to the nucleus by confocal microscopy. Vero cells were infected with WT or F170S HPIV1 at an MOI of 5 and, 48 h post-infection, were either mock-treated or treated with of IFN-β (1,000 IU/mL) for 60 min. Cells were then immunostained for the HPIV1 F and HN glycoproteins, to identify infected cells, and for Stat1 (Figure 3) or Stat2 (Figure 4). As expected, IFN-β treatment of mock-infected cells led to Stat1 translocation into the nucleus in the majority of treated cells (Figure 3). Similar results were observed following IFN-β treatment of F170S HPIV1-infected cells, showing that the F170S mutant virus was unable to inhibit translocation of Stat1 into the nucleus. In contrast, IFN-β treatment of WT HPIV1-infected cells was largely unable to induce Stat1 translocation to the nucleus, showing that WT HPIV1 effectively inhibited this step. While only 2% of WT HPIV1-infected cells stained positive for nuclear Stat1, 82% of the F170S HPIV1-infected cells stained positive for nuclear Stat1. For example, in the “WT+IFN” panel in Figure 3, Stat1 accumulated in the nuclei of three uninfected cells (right side) but not in any of the infected cells. Similarly, translocation of Stat2 to the nucleus in response to IFN-β was effectively inhibited by WT HPIV1 but not by F170S HPIV1 (Figure 4). While only 2% of WT HPIV1-infected cells stained positive for nuclear Stat2 following IFNβ treatment, 100% of the F170S-infected cells were positive for nuclear Stat2 (Figure 4). For example, Stat2 accumulated in the nuclei of two cells at the left of the “WT+IFN” panel in Figure 4, but these did not stain with the anti-HPIV1 antibodies and thus were uninfected.

### Co-immunoprecipitation of Stat1 and C′ protein

**Figure 5:**
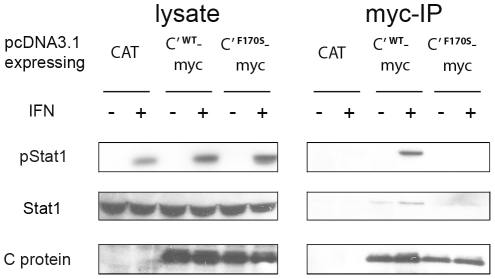
Co-immunoprecipitation of WT HPIV1 C protein and Stat1. 293 T cells were transfected with pcDNA3.1(+) plasmids expressing myc-tagged C′^WT^ or C′^F170S^ protein, or untagged CAT as a negative control. After 48 h, cells were mock-treated (IFN−) or treated (IFN+) with 1000 IU/ml of IFN-β for 30 min. Cell lysates were subjected to immunoprecipitation with anti-myc antibodies. Whole cell lysates and precipitates were separated on SDS-PAGE gels and analyzed by Western blot with antibodies against pStat1, Stat1, or the C protein, as indicated at the left. The experiment was carried out three times with comparable outcomes.

Since Stat1 and Stat2 were retained in the cytoplasm during infection with WT HPIV1 but not F170S HPIV1, we investigated whether retention might be due to physical interaction with the C proteins, as has been reported for SeV C proteins, and whether the C proteins interacted with both phosphorylated and unphosphorylated Stat proteins. Co-immunoprecipitation studies were performed using 293 T cells transfected with pcDNA3.1 plasmids expressing either myc-tagged C′^WT^ or C′^F170S^ protein, or untagged CAT protein as a negative control (Figure 5). This showed that, indeed, the C′**^WT^**-myc protein was able to co-immunoprecipitate both unphosphorylated and phosphorylated endogenous Stat1 (Figure 5, right panel). In contrast, the C′^F170S^-myc protein was unable to co-immunoprecipitate either form of Stat1 (Figure 5, right panel). We note that some co-immunoprecipiation of Stat1 was detected in untreated C′^WT^-myc transfected cells (fourth lane from the right), and that the amount of Stat1 co-precipitation was increased in IFN stimulated cells. Interestingly, the pStat1/Stat1 ratio was noticably higher in the precipitates (Figure 5, right panel) than in the lysates (left panel). This suggests that C′^WT^ proteins might bind pStat1 more efficiently than Stat1, although this has not been investigated further.

We also note that the level of Stat1 phosphorylation in the total lysates was not decreased in response to transfection with plasmid expressing either C′^WT^ or C′^F170S^ (Figure 5), We attribute this to the low transfection efficiency such that most cells did not express C′ protein and thus phosphorylation of most of the Stat1 in the culture would not be affected (not shown). In contrast, infection with WT or F170S HPIV1 was very efficient and resulted in a decrease in Stat1 phosphorylation that was evident in the total lysate (Figure 2). We also attempted to co-immunoprecipitate Stat2 with tagged C proteins but were unable to detect binding of C′^WT^ or C′^F170S^ to Stat2 (data not shown).

### Co-localization of Stat1 and HPIV1 C proteins

**Figure 6:**
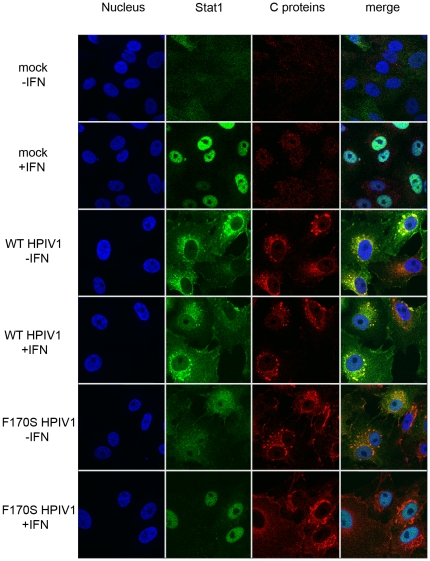
Co-localization of Stat1 and HPIV1 C proteins in Vero cells. Vero cells were mock-infected or infected with WT or F170S HPIV1 at an MOI of 1 TCID_50_/cell. After 48 h, cells were mock-treated (−IFN) or treated with 1000 IU/ml of IFN-β (+IFN) for 30 min. Cells were subsequently fixed, permeabilized, and stained for HPIV1 C proteins (red) and endogenous Stat1 protein (green). Z-stacks of Figure 6 are shown in the Videos S1, S2, S3, S4.

**Figure 7 pone-0028382-g007:**
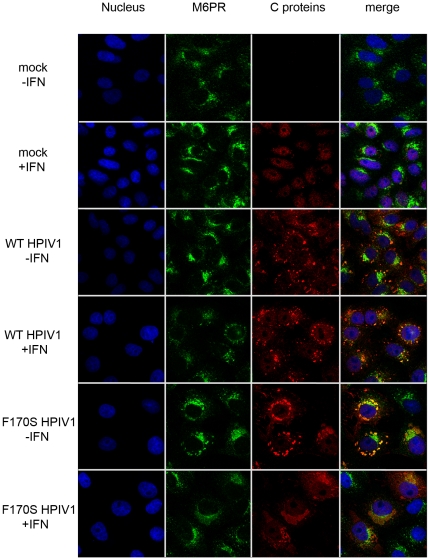
Co-localization of C proteins and mannose6-phosphate receptor in Vero cells. Vero cells were treated as described for Figure 6. Cells were stained for HPIV1 C proteins (red) and M6PR (green). Z-stacks of Figure 7 are shown in the Videos S5, S6, S7, S8.

We next examined the distribution of the HPIV1 C proteins and Stat1 in infected Vero cells using confocal microscopy. Vero cells were mock-infected or infected with WT or F170S HPIV1. Forty-eight h later, the cells were mock-treated or treated with 1000 IU/ml of IFN-β, and were fixed 60 min post-treatment and stained for HPIV1 C proteins (red) and endogenous Stat1 (green). Please note that the antiserum used to detect C proteins produces some background staining (cytosolic in mock-treated cells and nuclear in IFN treated cells [Figures 6 and 7, upper two rows]). In uninfected, untreated Vero cells, Stat1 was distributed evenly throughout the cytoplasm in a fine granular pattern (Figure 6). Upon IFN-β treatment, the Stat1 signal disappeared from the cytoplasm and concentrated in the nucleus. In cells infected with WT HPIV1 without subsequent IFN treatment, we observed that Stat1 was not distributed evenly, and instead accumulated around the nucleus in coarse perinuclear granules (Figure 6). In addition, in some infected cells a modest Stat1 accumulation signal was observed along the plasma membrane. In F170S-infected cells without subsequent IFN treatment, perinuclear Stat1 accumulation was also observed but formation of coarse granules was less distinct, and more of the Stat1 signal was evenly distributed throughout the cytoplasm. Following IFN treatment, the co-localization of Stat1 and C proteins in coarse perinuclear granules persisted in WT HPIV1-infected cells. In contrast, this co-localization disappeared completely in F170S HPIV1-infected cells and a strong Stat1 signal became visible in the nucleus (Figure 6). Although some of the coarse perinuclear granules in F170S-infected cells remained positive for C protein, they did not stain for Stat1, indicating that F170S C proteins were unable to retain Stat1 in these perinuclear granules and permitted translocation of Stat1 to the nucleus.

The perinuclear aggregates containing the C proteins and Stat1 that were observed in Figure 6 were less evident in Figure 3. This is because the photomicrographs in Figure 3 were taken at a higher z-plane (cross-section), largely above the intracellular location of the aggregates. With the use of a lower z-plane in Figure 6, the aggregates were readily and reproducibly detected. In order to visualize the three-dimensional distribution of the Stat1 and C signals in Figure 6, at least ten 0.17 µm cross-sections of infected cells (z-stacks) were acquired and a 3D reconstruction of the cells was performed (Videos S1, S2, S3, S4).

### Co-localization of the HPIV1 C proteins and Stat1, but not Stat2, with M6PR

**Figure 8 pone-0028382-g008:**
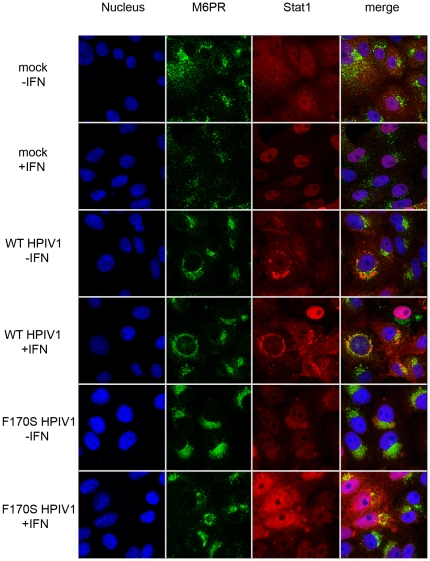
Co-localization of Stat1 and mannose6-phosphate receptor in Vero cells. Vero cells were treated as described for Figure 6. Cells were stained for Stat1 (red) and M6PR (green). Z-stacks of Figure 8 are shown in the Videos S9, S10, S11, S12.

**Figure 9:**
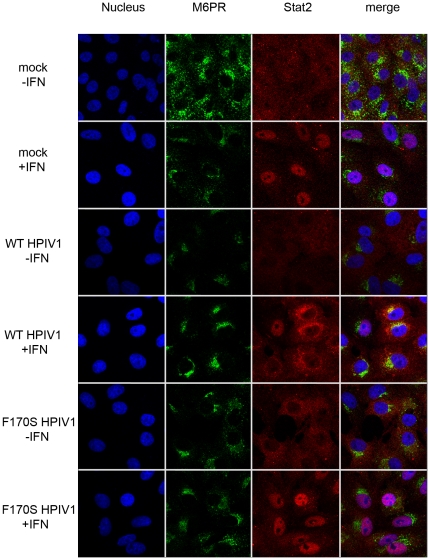
Co-localization of Stat2 and mannose6-phosphate receptor in Vero cells. Vero cells were treated as described for Figure 6. Cells were stained with for Stat2 (red) and M6PR (green). Z-stacks of Figure 9 are shown in the Videos S13, S14, S15, S16.

Aiming to identify the nature of these perinuclear granules in which Stat1 and C proteins co-localized, we stained infected cells with antibodies for a number of organelle-specific markers in addition to the C proteins and Stats. Whereas staining for endoplasmic reticulum (anti-protein disulfide isomerase [PDI]) or mitochondria (mitochondrial 60 K protein) yielded no overlapping signal (see Figure S3), staining using the late endosomal marker M6PR showed a high degree of co-localization with the HPIV1 C proteins (Figure 7 and Videos S5, S6, S7, S8) and Stat1 (Figure 8 and Videos S9, S10, S11, S12), but not Stat2 (Figure 9 and Videos S13, S14, S15, S16). These findings suggest that the HPIV1 C proteins associate with Stat1 on (or within) M6PR-positive vesicles, i.e., on or in late endosomes or intermediate vesicles from the trans-Golgi network. Specifically, the C proteins of both WT and F170S HPIV1 co-localized with M6PR both before and after stimulation with IFN-β. In the case of WT HPIV1, Stat1 also co-localized with M6PR both before and after stimulation. In the case of F170S HPIV1, Stat1 co-localized with M6PR before IFN-β stimulation, whereas afterwards it translocated to the nucleus. Stat2 appeared to be diffusely distributed in the cytoplasm of cells infected with either WT or F170S HPIV1, in contrast to the aggregated state of Stat1. Interestingly, following treatment of WT HPIV1-infected cells with IFN-β, Stat2 also appeared to aggregate in a perinuclear location (Figure 9). However, these aggregates did not form the dense granules that were often seen with Stat1, and these aggregates had less overlap with M6PR (Figure 9). In cells infected with F170S HPIV1, these aggregates were not observed following IFN-β treatment, and Stat2 accumulated in the nucleus, consistent with previous results. The Videos S1, S2, S3, S4, S5, S6, S7, S8, S9, S10, S11, S12, S13, S14, S15, S16 show the perinuclear granules and the co-localization or lack of co-localization in greater detail.

## Discussion

Inhibition of type 1 IFN induction, IFN signaling, and the establishment of an antiviral state are pivotal for efficient replication of HPIV1 and many other viruses [46]. We have previously shown that WT HPIV1 is able to suppress IFN-β induction and signaling, while F170S HPIV1 is unable to do so [25]. As a consequence, replication of F170S HPIV1 is restricted more than 100-fold in the respiratory tract of non-human primates [47]. In the present study, we took a closer look at the differences in IFN signaling between WT and F170S HPIV1, aiming to define at what step the virus-host interactions differ between these viruses.

We used African green monkey Vero cells for all of our assays except for the co-immunoprecipitation study, where 293 T cells were used because of their high transfection and protein expression efficiency. Vero cells are unable to express type 1 IFNs but are fully able to respond to exogenous IFN. Thus, one can evaluate IFN signaling in a controlled fashion by adding exogenous IFN without the confounding effects of endogenously produced IFN. This is particularly important because WT HPIV1 and F170S HPIV1 differ greatly in their ability to block IFN-β induction [25], which would complicate the distinction between effects on induction versus signaling. Vero cells also represent a susceptible host for HPIV1 infection. We also performed every experiment except the co-immunoprecipitation experiment in the context of viral infection rather than cDNA expression, which would provide an authentic environment for evaluating protein function and distribution. In Vero cells, infection with WT HPIV1 but not F170S HPIV1 inhibited the induction of an antiviral state, an indication of the extent of signaling following the addition of exogenous IFN-α, IFN-β, or IFN-γ. The level of restriction of VSV-GFP following IFN treatment was similar in uninfected versus F170S HPIV1-infected cells, indicating that this single point mutation essentially ablated the ability of the virus to inhibit signaling.

Although WT HPIV1 and WT SeV C proteins have previously been shown to block type 1 IFN signaling, most of the available information was for SeV, and it remained controversial where this block occurs (Introduction) [6], [26], [48]. Here, we did not observe a reduction in Stat1 or Stat2 accumulation in cells infected with WT or F170S HPIV1, in contrast to what is seen with Rubulavirus infection [45], [49], [50] (also see Figure 2). This is in agreement with previous reports on WT HPIV1 in human MRC5 cells [6]. For WT SeV, the situation is less clear, since the loss of Stat1 was observed in murine NIH 3T3 and BALB/c fibroblasts [35] but not in human HeLa or MRC5 cells [6], [51].

We also found that, in response to treatment with IFN-α, -β, and -γ, the accumulation of pStat1 and pStat2 was reduced in WT and F170S HPIV1-infected cells compared to mock-infected cells. Though WT HPIV1-infected cells showed marginally less phosphorylation for Stat2 than F170S HPIV1-infected cells, we were surprised to find that the F170S HPIV1 did not differ more drastically from WT HPIV1 in this regard. Thus we concluded that the inability of the F170S mutant to block signaling in response to IFN-α, -β, and -γ could not be explained at the level of phosphorylation of Stat1 and Stat2.

Following overnight exposure of Western blots, a small amount of pStat1 was detected in the absence of IFN treatment in WT HPIV1-infected cells, but not in F170S HPIV1-infected cells. A similar IFN-independent increase in pStat1 accumulation was previously reported for WT SeV and HPIV3 [51], [52], [53], [54]. WT SeV infection or expression of WT SeV C protein from transfected plasmid in HeLa cells also inhibited dephosphorylation of Stat1 [51]. Garcin et al. confirmed that neither Stat2, nor a functional IFN receptor, nor Jak1 were required for the SeV-mediated increase in pY701-Stat1 accumulation [55], supporting the idea that the increase in pStat1 resulted from virus-mediated inhibition of dephosphorylation, with the phosphorylation signal probably stemming from a background level of IFN-independent phosphorylation. Thus, our results suggest that HPIV1, like SeV, also inhibits dephosphorylation of Stat1. Since this activity was lost in F170S HPIV1-infected cells, it likely is a function of the HPIV1 C protein itself. While these observations further illustrate the greater Stat1 binding of WT C proteins versus F170S C proteins, this small amount of pStat1 present in the absence of IFN treatment likely does not contribute to inducing an antiviral state, since it is complexed with the C proteins.

Using fluorescence microscopy, we detected marked differences between WT and F170S HPIV1-infected Vero cells with regard to Stat1 and Stat2 translocation to the nucleus. WT HPIV1-infected cells remained negative for nuclear Stat1 and Stat2 following IFN-β treatment, but F170S HPIV1-infected cells permitted translocation of Stat1 and Stat2 to the nucleus. Our data for WT HPIV1 agree with results from Bousse et al. in MRC-5 cells [6], but F170S HPIV1 was not examined by these authors. The finding that a single amino acid substitution in C permits translocation strongly suggests that for WT HPIV1 the C protein is responsible for the observed block. We also found that WT C protein, but not the F170S C protein, could be co-immunoprecipitated with Stat1, as has been reported for SeV [35], [53]. Furthermore, WT C protein co-immunoprecipitated with both the phosphorylated and non-phosphorylated forms of Stat1, while co-immunoprecipitation with Stat2 was not detected. Additionally, the ratio of pStat1 to Stat1 was noticeably higher in the precipitates than in the lysates, suggesting that pStat1 was preferably bound by C′ protein. Such preferential binding of the phosphorylated form of Stat1 would be of interest, because it suggests that the C proteins preferentially target the active form of Stat 1. This also raises the possibility that the C proteins might bind to pStat1 contained in complexes such as with Stat2 and destabilize these complexes. However, further investigation using methods more suitable to measure binding affinity would be needed to investigate possible stronger association with pStat1.

Unexpectedly, we found that most of the Stat1 and C proteins in WT and F170S HPIV1-infected cells co-localized in fairly large perinuclear granules in the cytoplasm. While these complexes were observed with both viruses, the signal was somewhat less granular and dense with the F170S virus. Furthermore, for both viruses, these complexes largely co-localized with M6PR, which is a widely used marker for late endosomes. We believe this is the first report of the association of Respirovirus C proteins with large aggregates associated with the late endosome. Takeuchi et al. noted high molecular weight C protein:Stat1 complexes in SeV-infected cells based on size exclusion chromatography [53], but these complexes were not directly visualized in infected cells. In contrast to the present report, the SeV C proteins have generally been described as being associated with the plasma membrane. Marq et al. previously proposed that the SeV C proteins might be anchored to the plasma membrane by an amphipathic helix at the N-terminus of the C protein [56]. Also, Sakaguchi et al. reported co-localization of C proteins with Alix/AIP1 along the plasma membrane [57], suggesting that C proteins might recruit Alix to the plasma membrane to facilitate virus budding [22]. However, the significance of Alix for SeV budding is still controversial [24]. For HPIV1, most of the C protein and Stat1 protein in Vero cells infected with either the WT or F170S mutant appeared to be contained in these aggregates and not at the plasma membrane. Stat2 was distributed more evenly throughout the cytosol and in contrast to Stat1 did not seem to co-localize with M6PR. We note that two of the phenotypes that we do not detect, but which are described for SeV, namely Stat1 loss [36] and pronounced localization of either C proteins or Stat1 [56] to the plasma membrane, have both been ascribed to the N-terminal 23 amino acids of the SeV C′ protein, a region that is poorly conserved between HPIV1 and SeV.

The structure of the aggregates containing the C proteins, Stat1, and M6PR remains to be defined. Since the HPIV1 C proteins appear to lack a sequence for translocation across membrane, and since Stat1 quickly relocated to the nucleus in F170S HPIV1-infected cells following IFN treatment, it seems likely that the C protein:Stat1 complexes are located on the cytoplasmic face of late endosomes, rather than within the vesicles. Our microscopy data also suggests that the C protein might change the distribution of the late endosome. In non-infected cells, the late endosome looks polarized and sits like a cap on the nucleus. In contrast, in infected cells, distinct vesicles are frequently distributed all around the nucleus.

Stat2 did not appear to co-localize in these perinuclear aggregates, based on several observations. First, in the absence of IFN-β treatment, Stat2 appeared to be diffusely distributed in WT or F170S HPIV1-infected cells, in contrast to the Stat1 aggregates that clustered in the perinuclear space. Second, the Stat2-containing aggregates were not as well defined and not as dense as Stat1 aggregates. Third, these granules did not co-localize for the most part with M6PR. The finding that the Stat1-containing granules do not appear to contain Stat2 suggests that the C proteins bind predominantly to monomeric Stat1 rather than to the ISGF3 complex (Stat1:Stat2:IRF9). This suggestion is supported by the finding that Stat2 did not co-immunoprecipitate with C proteins, as would have been observed if the C proteins bound to ISGF3 complexes.

We previously tried to identify C protein binding partners using yeast-two-hybrid assays or immunoprecipitation, size-separation and mass-spectroscopy (unpublished data), but neither method identified Stat1 as a C protein binding partner. Only when the C′ protein (the largest form of the C proteins) was over-expressed in 293 T cells and the washing conditions for the immunoprecipitation were adjusted, could we co-immunoprecipitate Stat1 (and pStat1) protein with the WT HPIV1 C′ protein. Based on these findings, we suggest that the HPIV1 C proteins bind Stat1 (and pStat1) with only modest affinity to create an equilibrium that permits the binding partners to be exchanged and passed on frequently, and that a certain fraction of Stat1 proteins remains unbound at any time. Our studies suggest that the F170S C protein has an even lower affinity to Stat1 than does the WT C protein since it did not detectably immunopreciptate Stat1 and did not prevent Stat1 from entering the nucleus, thus permitting the establishment of an antiviral state. A higher affinity of WT C proteins towards Stat1, as compared to F170S C proteins, also was suggested by the detection of residual pStat1 in WT HPIV1-infected cells in the absence of IFN stimulation, whereas no pStat1 was detected in F170S HPIV1-infected cells in the absence of IFN stimulation, as already noted.

Depending on the particular virus, members of Paramyxovirinae may express both V and C (e.g., SeV; members of genus Morbillivirus [e.g. measles virus]; and members of Henipavirus [i.e., Nipah and Hendra viruses]), or only V (members of genus Rubulavirus [e.g. HPIV2] and Avulavirus [e.g., Newcastle disease virus]), or only C (HPIV1 and possibly HPIV3, as noted in the Introduction). Even though the C and V proteins are completely distinct, they can have similar effects in blocking host cell innate responses. However, the mechanisms involved can vary considerably between the two proteins and between different viruses, including the mechanisms for blocking signaling from the IFN-α/β receptor. As already noted, for SeV, IFN signaling appears to be blocked by the C proteins but not the V protein, involving inhibition of Stat phosphorylation and possibly degradation of Stat1 (Introduction). For the Rubulaviruses, the V protein was shown to promote degradation of Stat1 (parainfluenza virus 5 [49] and mumps virus [58]) or Stat2 (HPIV2 [50], also see Figure 2). For the Avulavirus Newcastle disease virus, the V protein inhibits IFN signaling by targeting Stat1 for degradation [59]. The V proteins but not the C proteins of measles virus inhibit IFN signaling by inhibiting Stat1 and Stat2 phosphorylation, but degradation of Stat1 or Stat2 was not observed [60], [61]. For Hendra and Nipah viruses, the V proteins inhibit signaling by binding to both Stat1 and Stat2, inhibiting their phosphorylation and creating cytoplasmic aggregates [62], [63]. Whether these Stat1 and Stat2 aggregates with the Henipavirus V proteins have any similarity to the aggregates between Stat1 and the HIPV1 C proteins reported in the present study is not known. Thus, there is little consistency with regard to the specific mechanisms associated with C or V or within most genera.

In summary, these studies showed that both the WT HPIV1 and the F170S mutant retain the ability to inhibit phosphorylation of Stat1 and, to a lesser extent, Stat2. Thus, the inability of the F170S mutant to block IFN signaling is not due to the loss of this ability. We found that the WT C proteins bind to Stat1 and pStat1 and sequester them in aggregates that co-localize with the late endosomal marker M6PR and are little affected by IFN treatment. This sequestration appears to be the mechanism by which the HPIV1 C proteins block signaling. Stat2 did not co-localize with M6PR or co-precipitate with C proteins, indicating that it was not contained in these aggregates. While the F170S C proteins retained the ability to aggregate Stat1 in perinuclear granules, they were unable to prevent nuclear translocation following IFN treatment. Co-immunopreciptation experiments indicated that this reflected lower-affinity binding due to the mutation. These results describe the mode of action of one of the major attenuating mutations present in a live attenuated HPIV1 vaccine candidate presently being evaluated in clinical trials (ClinicalTrials.gov ID NCT00641017).

## Materials and Methods

### Cells, Viruses and Plasmids

Vero cells (ATCC: CCL-81), LLC-MK2 cells (ATCC: CCL-7) and 293 T cells (ATCC: CRL-11268) were grown in OptiMEM (Gibco), supplemented with 10% fetal bovine serum (FBS). Infections with HPIV1 viruses were carried out using OptiMEM containing 1.2% Trypsin (Gibco) but no FBS. The recombinant F170S HPIV1 mutant was constructed in previous work to contain a phenylalanine-to-serine substitution at position 170 of the C protein but otherwise was confirmed by sequence analysis of the complete genome to be identical to its recombinant WT HPIV1 parent [26]. These viruses were grown in LLC-MK2 cells and purified on sucrose gradients as previously described to remove cytokines and other molecules derived from the infected cell [26]. VSV-GFP, a recombinant vesicular stomatitis virus expressing the green fluorescent protein from the first position in the genome, was grown in Vero cells [64]. The open reading frames of WT and F170S C′ protein (i.e., the longest version of the C protein) were PCR amplified and PCR products were cloned into pcDNA3.1(+) vectors. A pcDNA3.1(+) vector expressing untagged chloramphenicol acetyltransferase (CAT) protein was used as a negative control.

### VSV-GFP signaling assays

Vero cells were seeded into 6-well plates and two days later were infected with sucrose-purified WT or F170S HPIV1 at a multiplicity of infection (MOI) of 5 tissue culture infectious dose 50% (TCID_50_) per cell. 48 h later, cells were stimulated with 0, 100 or 1000 IU of IFN-α2a (Intron A, Schering), IFN-β1a (Avonex, Biogen) or IFN-γ (R&D, 285-IF/CF) for an additional 24 h. Cells were subsequently infected with 100 plaque-forming units of VSV-GFP per well and covered with overlay medium (OptiMEM+0.8% methylcellulose). Plaques were read 48 h later.

### Western Blots

Vero cells were seeded in 6-well plates and infected with sucrose-purified viruses at an MOI of 5. After 48 h of infection, cells were stimulated with 0 or 1000 IU of IFN-β1 for 30 min and subsequently lysed in 150 µl RIPA buffer (20 mM Tris-HCl, 100 mM NaCl, 1 mM EDTA, 1%NP-40, 1% Sodium deoxicholate, 0.1% SDS, pH 8.0 supplemented with protease and phosphatase inhibitors) per well. 10 µl of the lysates were separated on SDS-PAGE gels, blotted onto PVDF membranes, and probed with antibodies to phosphorylated (p)Stat1 (Y701) (Cell Signaling, #9171: 1∶1000), pStat2 (Y690) (Cell Signaling, #4441: 1∶500), Stat1 (Cell Signaling, #9172, 1∶1000), Stat2 (Santa Cruz, sc-464, 1∶300), PIV1 C proteins (Y2-35, polyclonal rabbit antiserum raised against the peptide TITTKTEQSQRRPK, which represents amino acids 79 to 91 in the C′ protein and thus reacts with all forms of C proteins, used at a dilution of 1∶2000), PIV2 P/V protein (mouse monoclonal, 85A, kind gift of Dr. Nishio [65] 1∶2000), and α-tubulin (Sigma, TG199, 1∶10000). Secondary antibodies from KPL (goat-anti-mouse-HRP, 074-1806 and goat-anti-rabbit-HRP, 074-1506) were used at concentrations of 100 ng/ml and bands were detected by chemiluminescence (Western Pico Detection Kit, Invitrogen) on Kodak Biomax MR films.

### Confocal Microscopy

Vero cells were seeded on cover slips in 24-well plates and were infected two days later with sucrose-purified viruses at an MOI of 1. After 48 h of infection, cells were stimulated with 1000 IU/ml of IFN-β1 for 1 h. Cells were subsequently washed with PBS, fixed with 2% paraformaldehyde in PBS for 10 min, permeabilized with 0.3% Triton X-100 in PBS for 10 min and then incubated in blocking buffer for at least 10 min (0.75% BSA+0.25% Gelatin in PBS). Cells were incubated with primary and secondary antibodies for 1 h each and washed with PBS three times. Cover slips were mounted on microscopy slides with DAPI-containing ProLong Gold reagent (Invitrogen). The following primary antibodies were used: anti-PIV1-C (rabbit, Y2-35, as above, 1∶1200), anti-PIV1 F/HN (1∶1 mixture of two mouse monoclonal antibodies against F and HN (F: 7.1; HN: 8.2.2.A [66]); 1∶1200), anti-Stat1 (rabbit, Santa-Cruz, sc-346, 1∶150), anti-Stat1 (mouse; Santa-Cruz; sc-464; 1∶600) and anti-Stat2 (rabbit; Santa-Cruz, sc-476,1∶150), and anti-cation independent mannose 6-phosphate receptor (M6PR, mouse, abcam, ab8093, 1∶400). The secondary antibodies were FITC-anti-mouse (Rockland, 810–1202, 3 µg/ml) and Alexa594 anti-rabbit (Invitrogen, A21207, 13 µg/ml). All antibodies were applied in 300 µl blocking buffer. Microscopy images were generated on a Leica Microsystems SP5 confocal microscope using a 63× oil immersion objective. For Figures 6 through [Fig pone-0028382-g007] [Fig pone-0028382-g008] 9 and the corresponding supplementary files an additional 3× zoom was applied to emphasize the intracellular structures. For quantification of Stat translocation to the nucleus, an investigator blinded to the treatment conditions examined 50 infected cells per read-out.

### Immunoprecipitation

Although all of the other experiments described above were carried out using Vero cells, these cells were difficult to transfect and did not express transfected plasmids efficiently. For this reason, we turned to 293 T cells for immunoprecipitation experiments. 293 T cells are 293 cells stably expressing the SV40 large T antigen, and plasmids containing the SV40 origin of replication are efficiently expressed. 293 T cells were seeded in 6-well plates and 2 days later they were transfected, using Lipofectamine 2000 (Invitrogen), with 5 µg of pcDNA3.1(+) per well, expressing either the WT or F170S C protein with a carboxyterminal myc-tag, or expressing untagged CAT protein (negative control). Cells were lysed in 600 µl lysis buffer (PBS+0.1% NP-40), 500 µl of which were incubated over night in a shaker with 20 µl of anti-C-myc agarose (Sigma, A7470). The slurry was washed twice with lysis buffer and proteins were eluted using 100 µl of 1xLDS Sample Buffer (Invitrogen, NP0008).

## Supporting Information

Figure S1 **Control experiments for the VSV-GFP assay used to quantify the IFN-induced antiviral state.** A) VSV-GFP plaque morphology. Vero cells were infected with sucrose gradient-purified WT or F170S HPIV1 at a multiplicity of infection (MOI) of 5 TCID_50_ per cell. 48 h later, cells were stimulated with 0, 100 or 1000 IU IFN-β1a (Avonex, Biogen) for an additional 24 h. Cells were subsequently infected with about 100 plaque-forming units of VSV-GFP per well and covered with overlay medium (OptiMEM+0.8% methylcellulose). Plaques were visualized 48 h later using a Molecular Dynamics Phosphorimager. B) GFP expression. Vero cells were seeded and infected similarly but the stimulation with 0, 100 or 1000 IU IFN-β1a was only 30 min. Cells were subsequently washed three times with PBS and infected with about 100 plaque-forming units of VSV-GFP per well. Afterwards OptiMEM without any supplements was added. Cells were lysed after 24 h and lysates were probed for GFP (abcam, ab290, 1∶500) and α-tubulin (Sigma, TG199, 1∶10000).
(TIF)

Figure S2 **Control experiment to show that the level of Stat1 phosphorylation following IFN stimulation is similar over time in WT HPIV1-infected versus F170S HPIV1-infected cells.** Vero cells were infected with WT HPIV1 or F170S HPIV1 or mock-infected at an MOI of 5. After 48 h of infection, cells were stimulated with 1000 IU/ml of IFN-β1 for multiple intervals. Cells were lysed in RIPA Buffer and 10 µl of the lysates were separated on SDS-PAGE gels, blotted onto PVDF membranes, and probed with antibodies to phosphorylated (p)Stat1 (Y701) (Cell Signaling, #9171: 1∶1000) and Stat1 (Cell Signaling, #9172, 1∶1000).
(TIF)

Figure S3 **The HPIV1 C proteins co-localize with the M6PR marker for late endosomes and not with markers for mitochondria or the endoplasmic reticulum.** Vero cells were infected with WT HPIV1 at an MOI of 0.5. After 48 h of infection, cells were stimulated with 1000 IU/ml of IFN-β1 for 1 h. Cells were subsequently washed with PBS, fixed with 2% paraformaldehyde in PBS for 10 min, permeabilized with 0.3% Triton X-100 in PBS for 10 min and then incubated in blocking buffer for at least 10 min (0.75% BSA+0.25% Gelatin in PBS). Cells were incubated with primary and secondary antibodies for 1 h each and washed with PBS three times. Cover slips were mounted on microscopy slides with DAPI-containing ProLong Gold reagent (Invitrogen). Primary antibodies were mouse-derived antibodies from abcam: ab3298 (mitochondria marker 1∶150), ab8093 (M6PR, late endosome marker, 1∶400), and ab2792 (anti-PDI, ER marker, 1∶200).
(TIF)

Video S1 **Supplemental video files for** **Figure 6** **(Stat1 and C proteins).** Vero cells were infected with WT HPIV1 for 48 h, mock-stimulated with medium containing neither FCS nor IFN-β for 1 h and subsequently fixed, permeabilized and stained for nucleus (DAPI, blue), Stat1 (green) and C proteins (red). Images of cross-sections (z-stacks) of 0.17 µm thickness were acquired on a Leica SP5 confocal microscope and 3D reconstructions were generated using Imaris software (Bitplane, Zurich, Switzerland). The first of the two rotations of the 3D reconstruction shows all three channels as indicated above, the second rotation shows the blue channel (DAPI, nucleus) and the “co-localization channel” (yellow, calculated from the co-localizing signals of the red and the green channels). Note that there is some cell-to-cell variability in expression: for example, the cell that is initially at the lower right expresses C proteins (red) but little Stat1 (green).
(MOV)

Video S2 **Supplemental video file for** **Figure 6** **(Stat1 and C proteins).** Vero cells were infected with WT HPIV1 for 48 h, stimulated with medium containing no FCS but IFN-β (1000 IU/ml) for 1 h and subsequently fixed, permeabilized and stained for nucleus (DAPI, blue), Stat1 (green) and C proteins (red). Images of cross-sections (z-stacks) of 0.17 µm thickness were acquired on a Leica SP5 confocal microscope and 3D reconstructions were generated using Imaris software (Bitplane, Zurich, Switzerland). The first of the two rotations of the 3D reconstruction shows all three channels as indicated above, the second rotation shows the blue channel (DAPI, nucleus) and the “co-localization channel” (yellow, calculated from the co-localizing signals of the red and the green channels).
(MOV)

Video S3 **Supplemental video file for** **Figure 6** **(Stat1 and C proteins).** Vero cells were infected with F170S HPIV1 for 48 h, mock-stimulated with medium containing neither FCS nor IFN-β for 1 h and subsequently fixed, permeabilized and stained for nucleus (DAPI, blue), Stat1 (green) and C proteins (red). Images of cross-sections (z-stacks) of 0.17 µm thickness were acquired on a Leica SP5 confocal microscope and 3D reconstructions were generated using Imaris software (Bitplane, Zurich, Switzerland). The first of the two rotations of the 3D reconstruction shows all three channels as indicated above, the second rotation shows the blue channel (DAPI, nucleus) and the “co-localization channel” (yellow, calculated from the co-localizing signals of the red and the green channels).
(MOV)

Video S4 **Supplemental video file for** **Figure 6** **(Stat1 and C proteins).** Vero cells were infected with F170S HPIV1 for 48 h, stimulated with medium containing no FCS but IFN-β (1000 IU/ml) for 1 h and subsequently fixed, permeabilized and stained for nucleus (DAPI, blue), Stat1 (green) and C proteins (red). Images of cross-sections (z-stacks) of 0.17 µm thickness were acquired on a Leica SP5 confocal microscope and 3D reconstructions were generated using Imaris software (Bitplane, Zurich, Switzerland). For better visibility of the nuclear localization of Stat1, the first of the two rotations of the 3D reconstruction shows the Stat1 (green) and C proteins (red) channels but not the blue channel (DAPI, nucleus). The second rotation shows the blue channel (DAPI, nucleus) and the “co-localization channel” (yellow, calculated from the co-localizing signals of the red and the green channels).
(MOV)

Video S5 **Supplemental video file for** **Figure 7** **(M6PR and C proteins).** Vero cells were infected with WT HPIV1 for 48 h, mock-stimulated with medium containing neither FCS nor IFN-β for 1 h and subsequently fixed, permeabilized and stained for nucleus (DAPI, blue), M6PR (green) and C proteins (red). Images of cross-sections (z-stacks) of 0.17 µm thickness were acquired on a Leica SP5 confocal microscope and 3D reconstructions were generated using Imaris software (Bitplane, Zurich, Switzerland). The first of the two rotations of the 3D reconstruction shows all three channels as indicated above, the second rotation shows the blue channel (DAPI, nucleus) and the “co-localization channel” (yellow, calculated from the co-localizing signals of the red and the green channels).
(MOV)

Video S6 **Supplemental video file for** **Figure 7** **(M6PR and C proteins).** Vero cells were infected with WT HPIV1 for 48 h, stimulated with medium containing no FCS but IFN-β (1000 IU/ml) for 1 h and subsequently fixed, permeabilized and stained for nucleus (DAPI, blue), M6PR (green) and C proteins (red). Images of cross-sections (z-stacks) of 0.17 µm thickness were acquired on a Leica SP5 confocal microscope and 3D reconstructions were generated using Imaris software (Bitplane, Zurich, Switzerland). The first of the two rotations of the 3D reconstruction shows all three channels as indicated above, the second rotation shows the blue channel (DAPI, nucleus) and the “co-localization channel” (yellow, calculated from the co-localizing signals of the red and the green channels).
(MOV)

Video S7 **Supplemental video file for** **Figure 7** **(M6PR and C proteins).** Vero cells were infected with F170S HPIV1 for 48 h, mock-stimulated with medium containing neither FCS nor IFN-β for 1 h and subsequently fixed, permeabilized and stained for nucleus (DAPI, blue), M6PR (green) and C proteins (red). Images of cross-sections (z-stacks) of 0.17 µm thickness were acquired on a Leica SP5 confocal microscope and 3D reconstructions were generated using Imaris software (Bitplane, Zurich, Switzerland). The first of the two rotations of the 3D reconstruction shows all three channels as indicated above, the second rotation shows the blue channel (DAPI, nucleus) and the “co-localization channel” (yellow, calculated from the co-localizing signals of the red and the green channels).
(MOV)

Video S8 **Supplemental video file for** **Figure 7** **(M6PR and C proteins).** Vero cells were infected with F170S HPIV1 for 48 h, stimulated with medium containing no FCS but IFN-β (1000 IU/ml) for 1 h and subsequently fixed, permeabilized and stained for nucleus (DAPI, blue), M6PR (green) and C proteins (red). Images of cross-sections (z-stacks) of 0.17 µm thickness were acquired on a Leica SP5 confocal microscope and 3D reconstructions were generated using Imaris software (Bitplane, Zurich, Switzerland). The first of the two rotations of the 3D reconstruction shows all three channels as indicated above, the second rotation shows the blue channel (DAPI, nucleus) and the “co-localization channel” (yellow, calculated from the co-localizing signals of the red and the green channels).
(MOV)

Video S9 **Supplemental video file for** **Figure 8** **(M6RP and Stat1).** Vero cells were infected with WT HPIV1 for 48 h, mock-stimulated with medium containing neither FCS nor IFN-β for 1 h and subsequently fixed, permeabilized and stained for nucleus (DAPI, blue), M6PR (green) and Stat1 (red). Images of cross-sections (z-stacks) of 0.17 µm thickness were acquired on a Leica SP5 confocal microscope and 3D reconstructions were generated using Imaris software (Bitplane, Zurich, Switzerland). The first of the two rotations of the 3D reconstruction shows all three channels as indicated above, the second rotation shows the blue channel (DAPI, nucleus) and the “co-localization channel” (yellow, calculated from the co-localizing signals of the red and the green channels).
(MOV)

Video S10 **Supplemental video file for** **Figure 8** **(M6RP and Stat1).** Vero cells were infected with WT HPIV1 for 48 h, stimulated with medium containing no FCS but IFN-β (1000 IU/ml) for 1 h and subsequently fixed, permeabilized and stained for nucleus (DAPI, blue), M6PR (green) and Stat1 (red). Images of cross-sections (z-stacks) of 0.17 µm thickness were acquired on a Leica SP5 confocal microscope and 3D reconstructions were generated using Imaris software (Bitplane, Zurich, Switzerland). The first of the two rotations of the 3D reconstruction shows all three channels as indicated above, the second rotation shows the blue channel (DAPI, nucleus) and the “co-localization channel” (yellow, calculated from the co-localizing signals of the red and the green channels).
(MOV)

Video S11 **Supplemental video file for** **Figure 8** **(M6RP and Stat1).** Vero cells were infected with F170S HPIV1 for 48 h, mock-stimulated with medium containing neither FCS nor IFN-β for 1 h and subsequently fixed, permeabilized and stained for nucleus (DAPI, blue), M6PR (green) and Stat1 (red). Images of cross-sections (z-stacks) of 0.17 µm thickness were acquired on a Leica SP5 confocal microscope and 3D reconstructions were generated using Imaris software (Bitplane, Zurich, Switzerland). The first of the two rotations of the 3D reconstruction shows all three channels as indicated above, the second rotation shows the blue channel (DAPI, nucleus) and the “co-localization channel” (yellow, calculated from the co-localizing signals of the red and the green channels).
(MOV)

Video S12 **Supplemental video file for** **Figure 8** **(M6RP and Stat1).** Vero cells were infected with F170S HPIV1 for 48 h, stimulated with medium containing no FCS but IFN-β (1000 IU/ml) for 1 h and subsequently fixed, permeabilized and stained for nucleus (DAPI, blue), M6PR (green) and Stat1 (red). Images of cross-sections (z-stacks) of 0.17 µm thickness were acquired on a Leica SP5 confocal microscope and 3D reconstructions were generated using Imaris software (Bitplane, Zurich, Switzerland). For better visibility of the nuclear localization of Stat1, the first of the two rotations of the 3D reconstruction shows the Stat1 (green) and C proteins (red) channels but not the blue channel (DAPI, nucleus). The second rotation shows the blue channel (DAPI, nucleus) and the “co-localization channel” (yellow, calculated from the co-localizing signals of the red and the green channels).
(MOV)

Video S13 **Supplemental video file for** **Figure 9** **(M6PR and Stat2).** Vero cells were infected with WT HPIV1 for 48 h, mock-stimulated with medium containing neither FCS nor IFN-β for 1 h and subsequently fixed, permeabilized and stained for nucleus (DAPI, blue), M6PR (green) and Stat2 (red). Images of cross-sections (z-stacks) of 0.17 µm thickness were acquired on a Leica SP5 confocal microscope and 3D reconstructions were generated using Imaris software (Bitplane, Zurich, Switzerland). The first of the two rotations of the 3D reconstruction shows all three channels as indicated above, the second rotation shows the blue channel (DAPI, nucleus) and the “co-localization channel” (yellow, calculated from the co-localizing signals of the red and the green channels).
(MOV)

Video S14 **Supplemental video file for** **Figure 9** **(M6PR and Stat2).** Vero cells were infected with WT HPIV1 for 48 h, stimulated with medium containing no FCS but IFN-β (1000 IU/ml) for 1 h and subsequently fixed, permeabilized and stained for nucleus (DAPI, blue), M6PR (green) and Stat2 (red). Images of cross-sections (z-stacks) of 0.17 µm thickness were acquired on a Leica SP5 confocal microscope and 3D reconstructions were generated using Imaris software (Bitplane, Zurich, Switzerland). The first of the two rotations of the 3D reconstruction shows all three channels as indicated above, the second rotation shows the blue channel (DAPI, nucleus) and the “co-localization channel” (yellow, calculated from the co-localizing signals of the red and the green channels).
(MOV)

Video S15 **Supplemental video file for** **Figure 9** **(M6PR and Stat2).** Vero cells were infected with F170S HPIV1 for 48 h, mock-stimulated with medium containing neither FCS nor IFN-β for 1 h and subsequently fixed, permeabilized and stained for nucleus (DAPI, blue), M6PR (green) and Stat2 (red). Images of cross-sections (z-stacks) of 0.17 µm thickness were acquired on a Leica SP5 confocal microscope and 3D reconstructions were generated using Imaris software (Bitplane, Zurich, Switzerland). The first of the two rotations of the 3D reconstruction shows all three channels as indicated above, the second rotation shows the blue channel (DAPI, nucleus) and the “co-localization channel” (yellow, calculated from the co-localizing signals of the red and the green channels).
(MOV)

Video S16 **Supplemental video file for** **Figure 9** **(M6PR and Stat2).** Vero cells were infected with F170S HPIV1 for 48 h, stimulated with medium containing no FCS but IFN-β (1000 IU/ml) for 1 h and subsequently fixed, permeabilized and stained for nucleus (DAPI, blue), M6PR (green) and Stat2 (red). Images of cross-sections (z-stacks) of 0.17 µm thickness were acquired on a Leica SP5 confocal microscope and 3D reconstructions were generated using Imaris software (Bitplane, Zurich, Switzerland). For better visibility of the nuclear localization of Stat1, the first of the two rotations of the 3D reconstruction shows the Stat1 (green) and C proteins (red) channels but not the blue channel (DAPI, nucleus). The second rotation shows the blue channel (DAPI, nucleus) and the “co-localization channel” (yellow, calculated from the co-localizing signals of the red and the green channels).
(MOV)
