# Supplementary figures and images for: The C Proteins of Human Parainfluenza Virus Type 1 Block IFN Signaling by Binding and Retaining Stat1 in Perinuclear Aggregates at the Late Endosome
Source: PLoS One. 2012 Feb 15;7(2):e28382. doi: 10.1371/journal.pone.0028382 (PMC3280236; doi:10.1371/journal.pone.0028382)

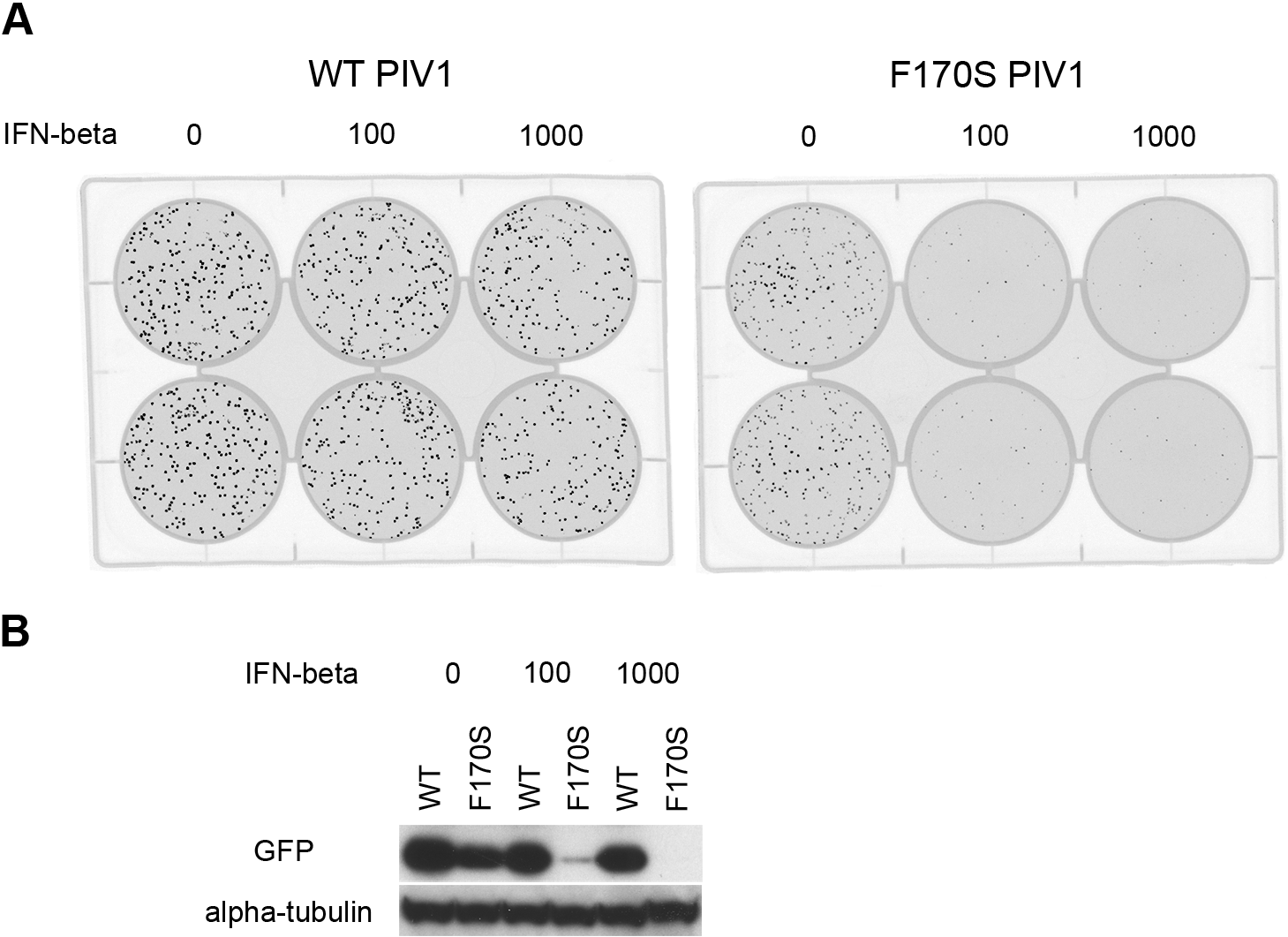

Supplement: Figure S1 — Control experiments for the VSV-GFP assay used to quantify the IFN-induced antiviral state. A) VSV-GFP plaque morphology. Vero cells were infected with sucrose gradient-purified WT or F170S HPIV1 at a multiplicity of infection (MOI) of 5 TCID50 per cell. 48 h later, cells were stimulated with 0, 100 or 1000 IU IFN-β1a (Avonex, Biogen) for an additional 24 h. Cells were subsequently infected with about 100 plaque-forming units of VSV-GFP per well and covered with overlay medium (OptiMEM+0.8% methylcellulose). Plaques were visualized 48 h later using a Molecular Dynamics Phosphorimager. B) GFP expression. Vero cells were seeded and infected similarly but the stimulation with 0, 100 or 1000 IU IFN-β1a was only 30 min. Cells were subsequently washed three times with PBS and infected with about 100 plaque-forming units of VSV-GFP per well. Afterwards OptiMEM without any supplements was added. Cells were lysed after 24 h and lysates were probed for GFP (abcam, ab290, 1∶500) and α-tubulin (Sigma, TG199, 1∶10000). (TIF) [file pone.0028382.s001.tif]

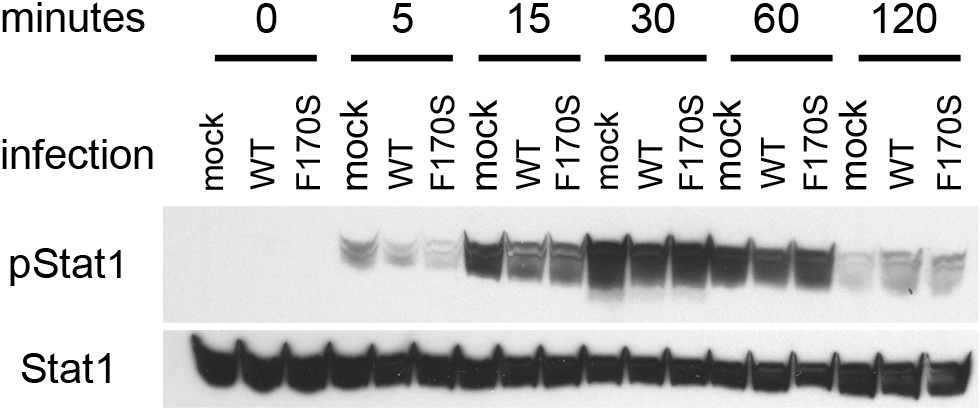

Supplement: Figure S2 — Control experiment to show that the level of Stat1 phosphorylation following IFN stimulation is similar over time in WT HPIV1-infected versus F170S HPIV1-infected cells. Vero cells were infected with WT HPIV1 or F170S HPIV1 or mock-infected at an MOI of 5. After 48 h of infection, cells were stimulated with 1000 IU/ml of IFN-β1 for multiple intervals. Cells were lysed in RIPA Buffer and 10 µl of the lysates were separated on SDS-PAGE gels, blotted onto PVDF membranes, and probed with antibodies to phosphorylated (p)Stat1 (Y701) (Cell Signaling, #9171: 1∶1000) and Stat1 (Cell Signaling, #9172, 1∶1000). (TIF) [file pone.0028382.s002.tif]

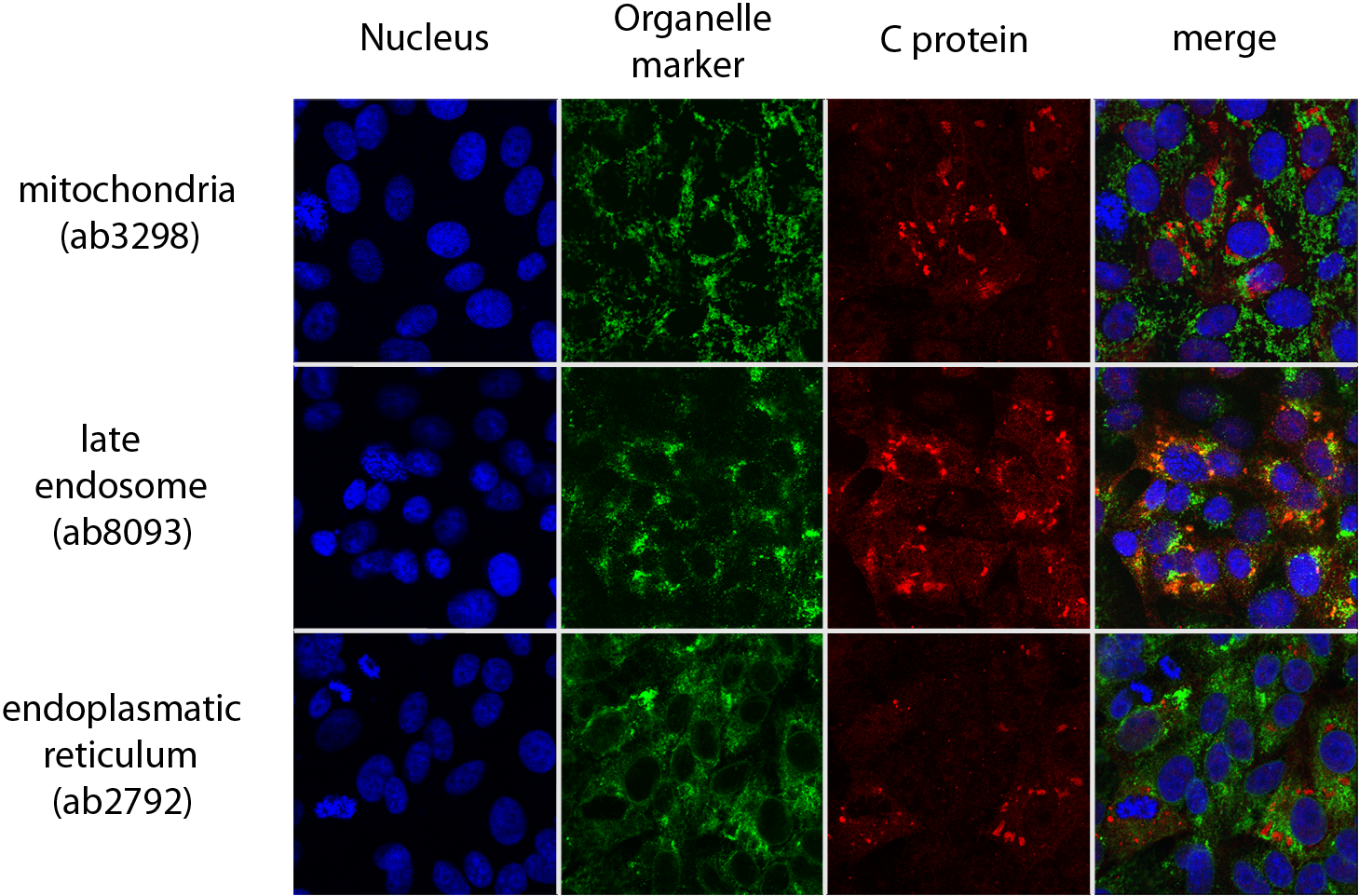

Supplement: Figure S3 — The HPIV1 C proteins co-localize with the M6PR marker for late endosomes and not with markers for mitochondria or the endoplasmic reticulum. Vero cells were infected with WT HPIV1 at an MOI of 0.5. After 48 h of infection, cells were stimulated with 1000 IU/ml of IFN-β1 for 1 h. Cells were subsequently washed with PBS, fixed with 2% paraformaldehyde in PBS for 10 min, permeabilized with 0.3% Triton X-100 in PBS for 10 min and then incubated in blocking buffer for at least 10 min (0.75% BSA+0.25% Gelatin in PBS). Cells were incubated with primary and secondary antibodies for 1 h each and washed with PBS three times. Cover slips were mounted on microscopy slides with DAPI-containing ProLong Gold reagent (Invitrogen). Primary antibodies were mouse-derived antibodies from abcam: ab3298 (mitochondria marker 1∶150), ab8093 (M6PR, late endosome marker, 1∶400), and ab2792 (anti-PDI, ER marker, 1∶200). (TIF) [file pone.0028382.s003.tif]
